# Distinguishing a Drone from Birds Based on Trajectory Movement and Deep Learning

**DOI:** 10.3390/s26030755

**Published:** 2026-01-23

**Authors:** Andrii Nesteruk, Valerii Nikitin, Yosyp Albrekht, Łukasz Ścisło, Damian Grela, Paweł Król

**Affiliations:** 1 Faculty of Informatics and Computer Engineering, National Technical University of Ukraine “Igor Sikorsky Kyiv Polytechnic Institute”, 37 Peremohy Ave., 03056 Kyiv, Ukraine; nesteruk.andrii@lll.kpi.ua (A.N.); nikitin.valerii@lll.kpi.ua (V.N.); albrekht.yosyp@lll.kpi.ua (Y.A.); 2 Faculty of Electrical and Computer Engineering, Cracow University of Technology, Warszawska 24, 31-155 Cracow, Poland; pawel.krol@pk.edu.pl

**Keywords:** unmanned aerial vehicles (UAVs), drone detection, motion trajectory analysis, long short-term memory (LSTM) networks, synthetic datasets, information technologies, computer vision

## Abstract

Unmanned aerial vehicles (UAVs) increasingly share low-altitude airspace with birds, making early distinguishing between drones and biological targets critical for safety and security. This work addresses long-range scenarios where objects occupy only a few pixels and appearance-based recognition becomes unreliable. We develop a model-driven simulation pipeline that generates synthetic data with a controlled camera model, atmospheric background and realistic motion of three aerial target types: multicopter, fixed-wing UAV and bird. From these sequences, each track is encoded as a time series of image-plane coordinates and apparent size, and a bidirectional long short-term memory (LSTM) network is trained to classify trajectories as drone-like or bird-like. The model learns characteristic differences in smoothness, turning behavior and velocity fluctuations, and to achieve reliable separation between drone and bird motion patterns on synthetic test data. Motion-trajectory cues alone can support early distinguishing of drones from birds when visual details are scarce, providing a complementary signal to conventional image-based detection. The proposed synthetic data and sequence classification pipeline forms a reproducible testbed that can be extended with real trajectories from radar or video tracking systems and used to prototype and benchmark trajectory-based recognizers for integrated surveillance solutions. The proposed method is designed to generalize naturally to real surveillance systems, as it relies on trajectory-level motion patterns rather than appearance-based features that are sensitive to sensor quality, illumination, or weather conditions.

## 1. Introduction

Unmanned aerial vehicles (UAVs) have rapidly evolved from niche platforms into widely accessible tools for logistics, inspection, photography and recreation [1]. At the same time, low-cost multirotor drones pose new risks to airports, critical infrastructure and public events, where unauthorized flights can cause safety incidents or be exploited for malicious purposes [2]. Moreover, UAVs are often a cause of problems for civilian infrastructure, due to issues with navigation in obstacle conditions like an urban environment [3]. In these contexts, automatic detection and classification of small UAVs is a key component of modern surveillance architectures, and computer vision (CV) plays an increasingly important role alongside radar, radio frequency (RF) and acoustic sensing [4]. A particularly important task is to reliably distinguish drones from birds, which frequently trigger false alarms and share the same low-altitude airspace [5,6].

However, robust drone–bird distinguishing in real deployments remains challenging. At long ranges a small target may occupy only a few pixels in each frame, making recognition based on appearance, texture or shape unreliable even for deep convolutional networks. Collecting and annotating large video datasets that contain diverse UAV types and bird species under varying weather, backgrounds and viewing geometries is logistically complex and often constrained by regulation. As a result, many CV-based systems are trained on relatively small or biased data, which limits their ability to generalize [7,8]. These difficulties motivate the use of simulation and synthetic data generation to explore algorithmic ideas, study edge cases and rapidly prototype classifiers before expensive field trials.

Previous research has mainly focused on several detection strategies that have been proposed to tackle the drone–bird problem. Some approaches focus on high-resolution imagery and attempt to exploit subtle shape or silhouette differences between drones and birds, which largely disappear at long distances [9]. Others combine CV with radar or RF sensors to improve robustness, but at the cost of system complexity [10]. A complementary line of work uses motion information, e.g., handcrafted trajectory descriptors (such as speed, acceleration, turning rate or wingbeat-induced jitter), or learned temporal features are used to separate biological and mechanical targets [11,12,13]. While promising, such methods often rely on limited proprietary datasets and lack a unified pipeline that ties together realistic motion models, camera geometry and a reproducible classification pipeline.

In operational surveillance, the primary requirement at the earliest stage is fast bird–drone discrimination to suppress false alarms: bird-like tracks can be ignored, while drone-like tracks are forwarded to subsequent response modules outside the scope of this study.

In this work, we investigate whether generated motion trajectories can provide a reliable cue for distinguishing drones from birds in long-range scenarios. To this end, we develop a model-driven simulation environment that generates synthetic data of three representative aerial targets: a multicopter, a fixed-wing UAV and a bird. The simulator incorporates a calibrated pinhole camera model, a parametric sky-and-cloud background and dynamic flight equations for each class, enabling us to control camera placement, field of view, distance to target and motion patterns in a consistent way. For each synthetic sequence, the target is detected and tracked, and its path is encoded as a time series of image plane coordinates and apparent size.

**Figure 1:**
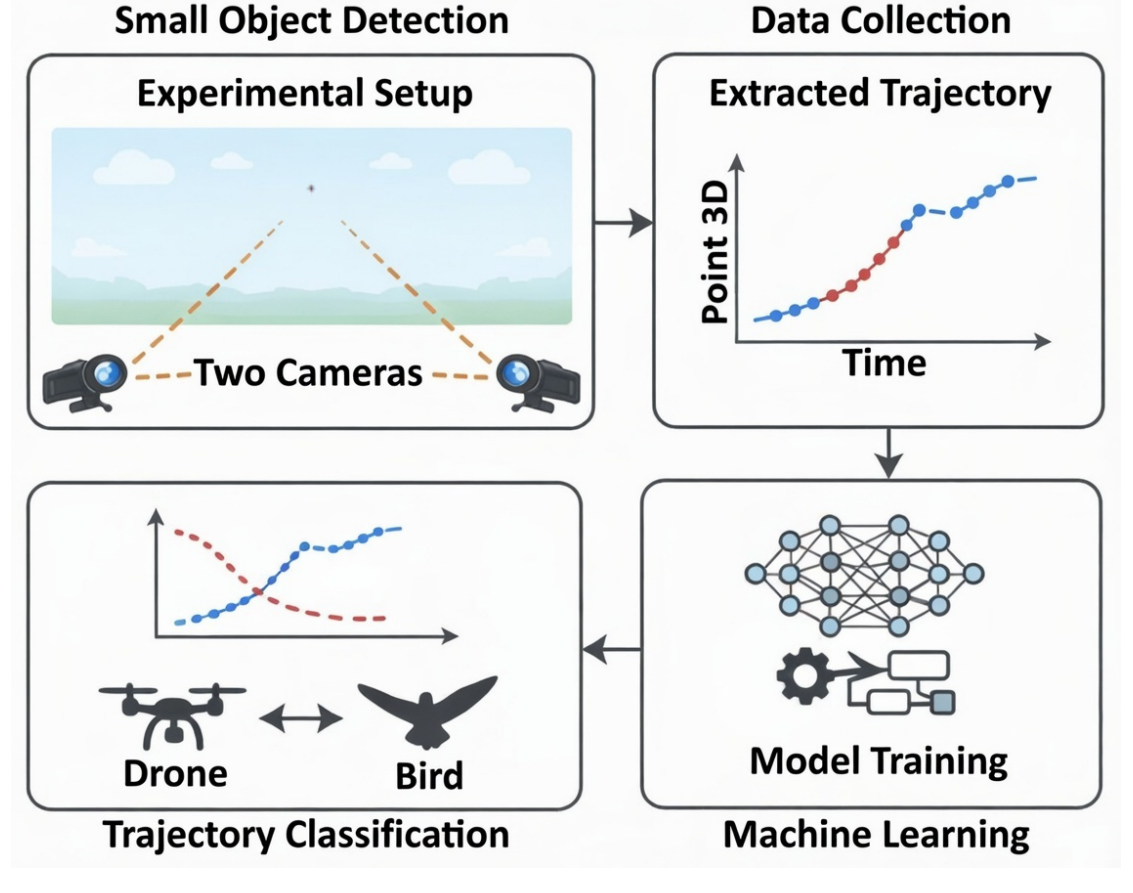
Distinguishing a drone from a bird; working principle.

On top of these trajectories, we build a compact deep-learning classifier based on bidirectional long short-term memory (LSTM) units, which is trained to assign each track to a “drone-like” or “bird-like” class. The aim of the research conducted by the authors was to verify whether the model is designed to capture differences in smoothness, maneuverability and local velocity fluctuations without relying on any handcrafted features beyond the raw position-and-size sequence. Additionally, the authors demonstrated that the experimental results on synthetic test data indicate that such a trajectory-based representation is sufficient to achieve clear separation between drone and bird motion patterns, suggesting that motion can serve as an effective early distinguishing signal when visual detail is scarce. The idea of the work principle is shown in Figure 1.

The contribution of this paper is threefold. First, we formulate bird–drone distinguishing as a trajectory-centric problem, targeting long-range scenarios where objects are observed as small blobs and appearance cues are weak or unavailable. Second, we introduce a physically and behaviorally motivated simulation model that generates annotated three-dimensional trajectories for multiple aerial target classes, enabling controlled and reproducible proof-of-concept evaluation when real 3D bird–drone trajectory datasets are scarce or inaccessible. Third, we demonstrate an end-to-end methodological pipeline from trajectory data extraction to temporal deep learning classification.

The rest of this paper is organized as follows: Section 2 reviews relevant work on drone and bird distinguishing, as well as trajectory-based approaches. Section 3 describes the proposed methodology, including the simulation environment, motion models and a trajectory-based deep learning classifier. Section 4 details the experimental setup and synthetic data generation procedure. Section 5 presents the experimental results. Section 6 discusses the obtained results, limitations and implications for practical surveillance systems. Finally, Section 7 provides conclusions and outlines future work directions.

## 2. Related Work

### 2.1. Methods for Distinguishing Birds from Drones

Research on unmanned aerial vehicle (UAV) detection spans a wide range of sensing modalities, including optical and infrared cameras, radar, radio frequency (RF) and acoustic sensors. Optical systems are attractive because they can leverage off-the-shelf cameras and recent advances in computer vision to detect and track small targets in cluttered scenes [6]. Radar-based and RF-based approaches, in turn, offer longer range and all-weather capability, but often struggle with very small radar cross sections and complex ground clutter, and typically require specialized hardware and regulatory compliance [14]. Hybrid systems that fuse optical, radar and RF information have been shown to improve robustness, but at the cost of increased system complexity and deployment overhead. Within this landscape, camera-based solutions remain a key component of many practical anti-drone systems, especially when fine-grained recognition of the target type is required [5,15].

A particularly challenging subproblem is the distinguishing of drones from birds. Appearance-based methods attempt to distinguish birds and multicopters using shape, silhouette, texture or high-resolution image patches. When resolution permits, convolutional neural networks (CNNs) trained on annotated images or video frames can exploit the presence of rotors, booms or fixed wings, as well as characteristic bird body and wing shapes. However, these cues degrade rapidly with distance: at long ranges the target occupies only a few pixels, and motion blur, atmospheric distortions and noise further obscure discriminative details. To address this, several works have proposed incorporating motion information [16,17,18]. Trajectory-based approaches compute features such as mean speed, acceleration, turning rate, trajectory smoothness or wingbeat-induced jitter, and feed these into classical classifiers or temporal models [19,20]. Birds typically exhibit more irregular, flapping-driven motion with sharper turns and vertical excursions, while drones tend to follow smoother, more constrained trajectories; nevertheless, quantitative modeling of these differences has been limited and often data-driven rather than model-driven.

The distinction between bird and UAV motion is closely related to their underlying flight dynamics. Multicopter drones are governed by attitude control loops that stabilize their orientation and enforce relatively smooth velocity profiles, with abrupt changes mainly associated with control commands. Fixed-wing UAVs follow curvilinear paths constrained by aerodynamic limits and minimum turning radii. Birds, in contrast, rely on flapping flight and complex maneuvers driven by environmental and behavioral factors, leading to richer short-term variability. Prior work on flight dynamics modeling [21,22,23,24] has largely focused either on UAV control and path planning or on biological flight studies, with only a small subset targeting the practical problem of drone–bird distinguishing in surveillance contexts. Existing operational systems often embed heuristic assumptions about smooth versus irregular motion rather than employing explicit generative models.

Deep learning has become the dominant paradigm for flying object recognition. Modern detectors and trackers based on CNNs and, more recently, transformer architectures have been applied to drone detection, bird monitoring and generic aerial target recognition. These systems can jointly localize and classify objects in each frame and are often extended with temporal modules—such as recurrent neural networks, temporal convolutional networks or attention mechanisms—to exploit motion cues across frames. For UAV–bird distinguishing, deep learning methods have been used both in image space (frame-wise classification of cropped patches) and in trajectory space (learning from sequences of positions or optical flow patterns) [16,18]. However, many reported models are trained on limited proprietary datasets, making it difficult to assess their robustness and to reproduce published results.

The scarcity of large, diverse and well-annotated datasets has motivated the use of synthetic data generation and simulation in computer vision for aerial targets. Several pipelines use game engines or model-driven renderers to simulate UAV flights, backgrounds, illumination and sensor characteristics, enabling controlled variation of scene parameters and systematic stress testing of algorithms [25]. Synthetic data have been successfully used to pre-train object detectors, augment training sets and study corner cases that are hard to capture in the field. Nonetheless, existing simulators for UAV detection often prioritize visual realism of the platform and environment, while motion trajectories are scripted or loosely specified rather than derived from unified flight and camera models for multiple target classes, including birds.

Early detection of small flying objects is an active research topic in both vision and radar communities. In optical systems, early detection is closely related to small object detection and track-before-detect strategies, where weak evidence is integrated over time before a firm decision is made. State-of-the-art methods attempt to detect targets at very low signal-to-noise ratios by combining spatial–temporal filtering, multi-frame integration and learned features. In this regime, appearance-based cues are minimal, and motion becomes a primary source of information. However, most early-detection studies focus on generic small objects or specific UAV types and rarely address systematic separation of drones from birds using motion alone [26,27].

Overall, previous works demonstrate that multimodal sensing and appearance-based recognition can be effective but, in the long-range recognition mode with few pixels, their reliability decreases, which motivates us to focus only on trajectory signals estimated in a supervised pipeline from modeling to classification.

### 2.2. Object Detection

Two-dimensional object detection is a computer vision task that involves detecting and localizing objects in an image in two-dimensional coordinates. Typically, the detection results in a bounding box around the location, the object class and its probability.

This detection does not provide information about depth (z) or 3D shape and is often used as the first stage before tracking: first the object must be detected in each frame, then it can be combined into a trajectory. One of the main difficulties is the inability to obtain the actual size in space, sensitivity to overlap and scale changes.

One of the classic 2D object detection algorithms is the histogram of oriented gradients (HOG) plus support vector machine (SVM), which was very popular before the advent of deep neural networks (YOLO, Faster R-CNN, etc.). It is still used for tasks that require speed and simplicity without large computational resources [28]. After receiving the image, features are extracted from it by analyzing the gradient directions using HOG. The resulting shape and texture of the image allows us to separate positive examples from negative ones. This method is effective for processing images with simple backgrounds and has a high processing speed but is very sensitive to overlaps and shape variations.

The Viola–Jones algorithm (2001) is one of the first successful detection methods that has found widespread use in real-time face detection in images [29]. It allows you to quickly discard most areas that definitely do not contain objects and spend more computation on only potential areas. During operation, simple features (Haar-like) are extracted, and a small number of the most discriminative features are selected, which then enter the cascade classifier. This method is very fast, thanks to the integral image and cascade, but is limited in generalization and sensitive to variations in lighting, angle and partial overlaps. Although the algorithm is a general pipeline for object detection, the variability of shapes and poses, the influence of lighting, background and different scales and distances make it unsuitable for the effective detection of birds and drones.

In the context of long-range detection, when a flying object is a small number of pixels and has some contrast, it makes sense to use so-called blob detectors. LoG (Laplacian of Gaussian) and DoH (determinant of Hessian) belong to the class of “blob detectors” and keypoint detectors [30,31]. They search for image features based on derivatives, gradients or curvature. The determinant of Hessian method is based on the analysis of the Hessian matrix, which describes the curvature of the image. Using second derivatives, it allows you to find stable points in the scale space, and local extrema of the Hessian determinant correspond to “blobs”. This method is the basis of the SURF algorithm and is more sensitive to complex structures than LoG or DoG.

The Harris corner detector algorithm is based on gradient computation and estimates how much intensity changes in two directions. If the changes are strong in both directions, the point is defined as a “corner” [32]. In the context of drone and bird detection, Harris is good at finding corners of the fuselage or wing edges, although it is not scale invariant.

Shi–Tomasi is an improvement on the Harris method. It suggests selecting keypoints based on the magnitude of the eigenvalues of the autocorrelation matrix, which allows for better identification of “good” points for tracking [33]. This approach is often used in conjunction with the optical flow method for tracking objects in video.

Features from accelerated segment test (FAST) was designed to work quickly in real time. The method checks the intensities of pixels in a circle around a candidate: if a certain number of neighbors are significantly brighter or darker than the central pixel, that point is considered a keypoint. This allows you to find corners very quickly [34].

Maximally stable extremal regions (MSER) looks for stable regions in an image that remain similar when the binarization threshold is changed. This allows for the detection of high contrast regions, such as a dark drone in a bright sky. The algorithm is known for its robustness and is widely used for text detection, but it can also be used for object tracking in complex scenes [35].

Scale-invariant feature transform (SIFT) is one of the most famous computer vision algorithms. It uses DoG to find extrema in a scale space and then describes each keypoint using gradient descriptors. SIFT is scale invariant, rotation invariant and partially illumination invariant, making it extremely robust in complex environments [36].

Speeded-up robust features (SURF) was proposed as an optimization of SIFT. It uses the Hessian determinant in combination with integral images and box filters, which allows for significant computational speedup without loss of robustness. SURF works well for detecting keypoints in images with large variations in scale and illumination [37].

Therefore, instead of proposing a new detector, this study considers object detection and tracking as a preprocessing step to obtain trajectories on a noisy image plane and focuses on how these trajectories can support the distinguishing of drones and birds when appearance information is limited.

In summary, prior work has demonstrated the potential of multi-sensor systems, deep learning and motion analysis for drone detection, but also highlighted limitations, e.g., a lack of open, balanced datasets for UAV–bird distinguishing; limited exploitation of unified, model-driven motion models across multiple aerial target types; and sparse evaluation of purely trajectory-based classifiers in realistic long-range scenarios. The present study builds on these lines of research by combining a controlled data simulation environment with deep sequence modeling, and by focusing explicitly on the question of how far motion trajectories alone can be pushed to distinguish drones from birds when appearance information is severely constrained.

### 2.3. Object Tracking and Association

#### 2.3.1. Data Association Between Frames

Data association is a classic problem in computer vision that consists of matching points/objects in a sequence of frames. The main approaches and methods include optical flow-based, state filtering, data assignment methods, descriptor-based tracking and multi-object tracking pipelines.

Optical flow is a vector field that describes an approximation of the movement of pixel brightness between two adjacent video frames. The idea is simple: if an object moves, its image shifts and we can estimate this shift:

I(x, y, t) = I(x + delta x, y + delta y, t + delta t),(1)

where I(x, y, t) is intensity at point (x, y) at time t.

From here the optical flow constraint equation (OFCE) is derived:

I_x_u + I_y_v + I_t_ = 0, (2)

where I_x_, I_y_ are spatial derivatives, I_t_ is the time derivative, (u, v) are components of the flow (velocity) vector.

The Lucas–Kanade algorithm [38] is used to assume the motion of all pixels between two consecutive images. This method is applicable in the model presented above. Thus, instead of solving optical flow Equation (1) for a single pixel (which is an insufficient problem), a redundant system of equations for several neighboring points can be obtained. This system is solved by the least squares method, which allows us to calculate the velocity vector for the center of the window. Lucas–Kanade works well for small object movements and for textured areas where the brightness changes in different directions. It is not a global method but rather a local one—it estimates the motion in certain areas. The algorithm is still used today in OpenCV and is especially effective in combination with multiscale pyramids, which allows us to find large movements.

A more recent line of work extends global/variational optical flow formulations to cases where the brightness constancy assumption is violated by motion blur. For example, Daraei [39] introduced MB-CLG, a blur-aware scheme that first constructs a consistent pair of blurred frames (to account for spatially varying blur kernels) and then applies a standard dense optical flow computation. The method follows a coarse-to-fine optimization strategy and incorporates a smoothness matrix to better handle occlusions; moreover, instead of warping image frames or precomputing large derivative grids, it warps the flow estimate at each iteration, reducing both storage and computational overhead. As with classical global approaches, this regularized formulation produces dense per-pixel motion fields and suppresses noise, but its accuracy still depends on the adequacy of the adopted blur model and can degrade when the assumed blur/appearance formation does not match the true scene dynamics.

In 2003, Gunnar Farnebäk [40] proposed an algorithm that has become the standard for estimating dense optical flow in OpenCV. His idea is based on approximating local regions of an image with quadratic polynomials. He then evaluates how these approximations shift between frames. This approach allows not only to track individual points but also to build a map of motion vectors for the entire image. Farnebäk’s method turned out to be more robust to noise and faster than the classical Horn–Schunck, so it is actively used in practical systems for motion analysis.

The Lucas–Kanade algorithm in its basic form works well only for small displacements. To extend its application to large displacements, a multiscale extension using an image pyramid was developed. The idea is to build a hierarchy of copies of an image at different resolutions, from greatly reduced to the original. First, the motion is estimated at the coarsest level (where the displacements are smaller in pixel coordinates), after which the result is refined at higher resolution levels. Thus, the Lucas–Kanade pyramid combines the speed and simplicity of the original algorithm with the ability to track large displacements, making it one of the most practical options in real-world computer vision systems.

With the development of deep learning, methods have emerged that allow estimating optical flow directly from data rather than through manual models. The first known example was FlowNet by Dosovirskiy et al. [41], where a convolutional neural network was trained to predict motion vectors between two frames. This approach showed that, even without complex optimization variations, competitive results can be obtained. Later, PWC-Net by Sun et al. [42] appeared, which introduced a pyramidal structure, correlation calculations and context blocks, which significantly increased accuracy with a relatively small number of parameters. The most modern and accurate method is RAFT [43], which uses recurrent architectures and multiple refinement of the motion field based on global pairwise comparisons. Deep methods outperform classical approaches in terms of accuracy and robustness, especially in difficult conditions with large displacements, non-stationary illumination or strong deformations of objects.

In tracking, the state estimate is refined by combining a motion prediction with measurement updates from the detector. This filtering step reduces jitter and compensates for occasional missed or noisy detections.

#### 2.3.2. Object Tracking

In object tracking problems, we deal with a dynamic system. The object moves in space and its position changes from frame to frame. But we never see the true trajectory—we only have noisy measurements (e.g., the centroid of the blob in each frame, which can jump due to sensor noise or detector errors).

Formally, the system is described as:

- State model (dynamics):
  x_t_ = F_xt−1_ + B_ut_ + w_t_, (3)
  where x_t_ is state vector (e.g., position and velocity); F is the state transition matrix; u_t_ is the control input; w_t_ is the noise.
- Observation model:
  z_t_ = H_xt_ = v_t_, (4)
  where z_t_ is the measurement (for example, the coordinate of the object in the frame); H is the projection matrix; v_t_ is the measurement noise.

The classical algorithm, proposed by R. Kalman [44], is used for systems with linear dynamics and Gaussian noise. It cyclically performs two steps:

- Prediction: Based on the previous state, a new state *xt* and the uncertainty covariance are estimated.
- Update: The received measurements are compared with the prediction, the difference (residual) is calculated and the state is adjusted taking into account the weight (Kalman gain).

In cases where the object’s motion is nonlinear (for example, a drone changes trajectory along a curved trajectory), the classical Kalman filter does not work directly. In this case, a linear approximation (Taylor expansion) around the current state estimate is applied. This method is called the extended Kalman filter. It works well for weakly nonlinear systems but can give errors if the nonlinearity is strong.

For very complex cases, where neither linearity nor Gaussian assumptions hold, particle filters are used. In this approach, the state of the system is represented not by a single estimate and covariance but by a set of random samples (particles), which are distributed according to a probability distribution.

Each particle models the possible position and velocity of the object, and when new measurements are received, the particle weights are updated and resampled. In this way, even very complex trajectories with noise and sharp maneuvers can be tracked.

Particle filter is often used in robotics when a drone or mobile robot moves in space with unpredictable turns and obstacles. The Kalman filter is well-suited for uniform flight (for example, when the drone flies straight). Extended Kalman or particle filter will be needed when the trajectory is complex and the predictability is low. In practical systems, the state filter is often combined with data association algorithms (Hungarian algorithm, Deep SORT) to solve the problem of prediction and matching of multiple targets simultaneously [45].

An important point during frame processing is data association. There are situations when it is necessary to match objects from the previous frame to objects in the current frame. This allows you to track which object continues its trajectory and allows to build continuous trajectories of objects in time. Such methods include nearest neighbor (NN), Hungarian algorithm (Kuhn–Munkres), joint probabilistic data association (JPDA), multiple hypothesis tracking (MHT) and association with appearance features.

The nearest neighbor method is the simplest way to solve the data association problem. The idea is that each object from the previous frame is assigned the object from the new frame that is closest in distance. The distance is usually measured by the Euclidean distance, sometimes by the Mahalanobis distance or by other metrics. This approach works well when there are few objects, the scene is relatively simple and the objects move smoothly and do not intersect. However, in complex conditions, it is prone to errors, e.g., if two birds fly side by side, the algorithm can confuse their trajectories by assigning the wrong neighbor. Therefore, the nearest neighbor method is suitable as a basic method, or for tasks with one or two objects, but does not provide reliability for multi-contact tracking.

In more complex problems, the Hungarian algorithm (Kuhn–Munkres) is used, which is a classic method for solving the so-called assignment problem. In this case, we build a cost matrix, where each row corresponds to an object from the previous frame and each column to an object from the current one. An element of the matrix shows the “cost” of the correspondence between two objects, usually a distance or a difference in other parameters. The algorithm finds a matching that minimizes the total cost for the entire scene. This avoids local errors inherent in the nearest neighbor method and guarantees a globally optimal solution. In drone and bird tracking, the Hungarian algorithm is used very often, especially in combination with state filters (e.g., Kalman), where the cost matrix is built on the basis of predicted positions [46].

The method of probabilistic data association (JPDA) goes further and considers all possible assignments between measurements and tracks. Instead of choosing one “best” match, it calculates the probability of each possible association and forms a new state estimate as a weighted average. This is especially useful when several objects are close to each other and it is not possible to unambiguously determine which measurement corresponds to which object. JPDA is often used in surveillance and aviation systems for tracking aircraft or drones, where accuracy is critical. In the problem of tracking birds in a flock, JPDA allows to avoid confusion when the objects almost overlap and the detector gives ambiguous observations [47].

An even more powerful approach is multiple hypothesis tracking (MHT). In this method, instead of immediately deciding which assignment is correct, the system maintains several alternative hypotheses and evolves them over time. As more data become available, the less likely hypotheses are discarded while the more likely ones are retained. This approach is especially valuable in very complex environments where trajectories often intersect or objects disappear and reappear. For example, if several drones simultaneously fly past trees and disappear from view for a few seconds, MHT allows you to store several options for which will appear where, and then refine the choice. The main disadvantage of MHT is its high computational cost, as the number of hypotheses grows exponentially with the number of objects and time [48].

Modern tracking methods increasingly use not only the coordinates of objects but also their visual appearance. The idea is to calculate for each object a set of descriptors (appearance features), for example, feature vectors obtained using convolutional neural networks. Then, the association between objects in different frames is performed not only based on the distance in space but also on the similarity of their appearance. This is especially useful when objects intersect or move in dense groups. In the case of birds, the shape of the silhouette or the texture of feathers can be analyzed, and for drones, the characteristic features of the body or propellers. One of the most popular algorithms in this class is Deep SORT, which combines the Kalman filter for motion prediction with a neural network for assessing the similarity of objects. Thus, the system is able to track dozens and even hundreds of objects in real time with high accuracy [49].

In summary, the existing literature on drone detection demonstrates high performance when clear appearance cues or additional sensing techniques are available, but it remains poorly effective for long-range conditions where targets occupy only a few pixels and visual details disappear. Moreover, progress is hampered by the limited availability of open and balanced datasets on UAVs and birds, and by the fact that many motion-based studies rely on proprietary recordings or highly specialized scenarios. As a result, there is a practical gap in reproducible, comprehensive assessments that combine realistic target motion, camera projection and a trajectory-only classifier under controlled but varied conditions. This study addresses this gap by using a controlled pipeline from modeling to classification and investigating the extent to which trajectory cues (image plane motion and apparent size over time) can distinguish drones from birds when appearance information is severely limited. To address this gap in a reproducible manner, Section 3 (Materials and Methods) describes our pipeline from simulation to classification, including a synthetic data generation environment, motion models, image plane trajectory encoding and an LSTM-based classifier used in subsequent experiments.

## 3. Materials and Methods

This section describes the simulation environment, the flight dynamics models for different aerial targets, the procedure for synthetic data generation and the deep learning pipeline used to classify motion trajectories as “drone-like” or “bird-like”.

**Figure 2:**
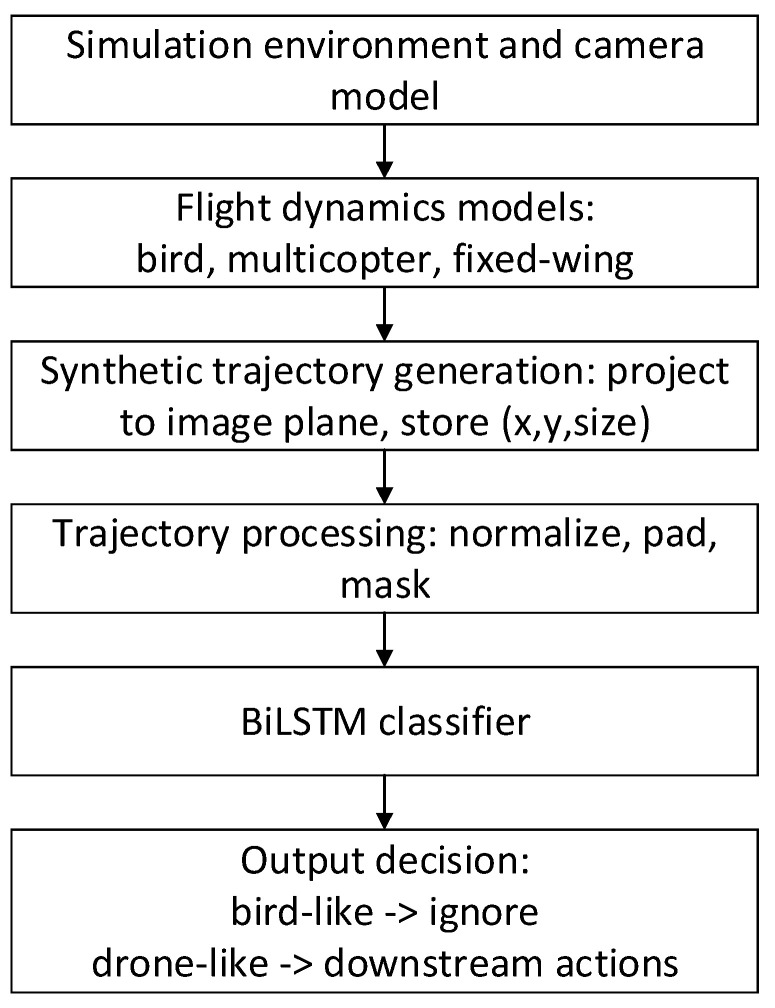
Drone–bird classifier diagram.

To improve the readability of the proposed multi-stage procedure, Figure 2 summarizes the overall pipeline used in this study. The workflow starts with a physics-based simulation of aerial target motion and scene geometry (Section 3.1 and Section 3.2), followed by synthetic trajectory generation and representation in the image plane (Section 3.3). Next, trajectories are preprocessed (normalization and padding/masking) and passed to the bidirectional LSTM classifier (Section 3.4), which outputs a binary decision (“bird-like” vs. “drone-like”) used as a first-stage filter in surveillance scenarios. Each block in Figure 2 corresponds directly to the subsections of Section 3.

### 3.1. Simulation Environment

#### 3.1.1. Camera Model and Environment Formation

The experiments use a synthetic data environment that mimics a stationary, upward-looking camera observing small flying objects against the sky. The camera is modeled as an ideal pinhole camera without lens distortion or rolling shutter effects. Its projection model maps 3D world points to 2D image coordinates via a standard calibration matrix and rigid transform [50].

K = [[f_x_, s, c_x_], [0, f_y_, c_y_], [0, 0, 1]], (5)

where f_x_, f_y_ are the focal lengths in pixels along the horizontal and vertical axes, c_x_, c_y_ are the principal points (optical center) in pixels, s is the skew factor (typically 0 for square pixels).

The equivalent focal length f (in pixels) used to set f_x_ and f_y_ is derived from the desired field of view (FOV) and image width N_px_ as [51]:

f = (N_px_/2)/tan(FOV/2), (6)

where N_px_ is the size of the matrix (in pixels) along the corresponding dimension.

With this camera configuration, an object of known physical size at a given distance projects to a predictable number of pixels in the image plane.

Throughout this work the camera is assumed to be rigidly fixed in space and oriented upwards; all apparent motion in the image is therefore due only to the movement of the simulated targets.

The background is modeled as an open sky with a vertical color gradient and slowly drifting semi-transparent clouds [52]. A base sky image is generated as a 2D array of RGB pixels of size H × W. For each row y (with y = 0 at the top and y = H − 1 at the bottom), we linearly interpolate between a zenith color C_top and a near-horizon color C_bottom:

alpha = y/(H − 1),(7)

C_sky(y) = (1 − alpha) ∗ C_top + alpha ∗ C_bottom.(8)

This produces a darker blue at the top of the frame and a lighter, almost white tone near the bottom, imitating atmospheric haze and overexposed horizon.

Clouds are represented by a time-dependent opacity mask M(x, y, t) with values in [0, 1]. At the initial time we generate several Gaussian blobs with random centers and radii, sum them to obtain a base mask, then normalize and attenuate it towards the horizon so that distant clouds appear thinner. To simulate motion, the blob centers are shifted slightly from frame to frame according to slow sinusoidal functions of time (horizontal and vertical drift). At each simulation step, the final image is obtained by alpha blending the sky gradient with a constant cloud color (near white) using the mask [53]:

I(x, y, t) = (1 − alpha_cloud ∗ M(x, y, t)) ∗ C_sky(y) + alpha_cloud ∗ M(x, y, t) ∗ C_cloud,(9)

where alpha_cloud is the maximum cloud opacity (e.g., 0.5–0.6). Because the camera is looking strictly upwards and the ground is not modeled, every frame consists solely of this dynamic sky background with superimposed flying objects rendered at their projected positions and apparent sizes. This environment provides a controlled but visually plausible setting for studying long-range trajectories of drones and birds.

#### 3.1.2. Multicopter

A quadcopter is a multirotor aircraft with four propellers generating thrust and torques for control. We define a body-fixed frame at the vehicle’s center of mass, with axes aligned such that the thrust from each rotor points along the body’s *z*-axis (upward). The orientation can be described by Euler angles: roll φ, pitch θ and yaw ψ. The transformation from body rates (p, q, r) to Euler angle rates ($\dot{\varphi }$, $\dot{\theta }$, $\dot{\psi }$) is given by a matrix W(φ, θ), ensuring we can relate angular velocity in the body frame to the time derivatives of φ, θ and ψ.

The total thrust T produced by the four rotors acts along the body’s *z*-axis and opposes gravity. In the inertial (world) frame, the quadcopter’s acceleration x″ = (x″, y″, z″) is governed by the balance of thrust and weight. Using the rotation matrix R(φ, θ, ψ) from body to inertial frame (so that R[0, 0, 1]^T^ is the body’s up-axis in world coordinates), we have:

mx″ = R(φ, θ, ψ) [0, 0, T] − mg [0, 0, 1],(10)

which indicates that the thrust contributes to acceleration in the direction of the body’s *z*-axis and gravity mg acts downward. Here g is gravitational acceleration, m is mass of quadcopter and T is the total thrust force equal to the sum of forces f_Mi_ from each motor i in Equation (12).

Each rotor i produces an upward force f_Mi_ approximately proportional to the square of its angular speed ω. We can write the thrust model for each rotor as:

f_Mi_ = k_t_ω_i_, (11)

where k_t_ is a thrust coefficient relating motor speed to generated force. The total thrust is then:

T = sum_{i = 1…4} f_Mi_ = k_t_ ∗ (ω_1_^2^ + ω_2_^2^ + ω_3_^2^ + ω_4_^2^), (12)

This net thrust T appears in the translational equation above and is the primary control for altitude and vertical acceleration [54].

The rotational motion is described by Euler’s equations for a rigid body. In vector form, the angular velocity of the quadcopter in body frame is ω = (p, q, r) and it evolves according to:

I ω^′^ + ω × (I ω) = τ, (13)

where τ = (τ_φ_, τ_θ_, τ_ψ_) is the vector of total moments about the body’s x, y and z axes. Expanding this equation in components yields the set of nonlinear equations:

I_xx_ p^′^ = (I_yy_ − I_zz_)qr + τ_φ,_ (14)

I_yy_ q^′^ = (I_zz_ − I_xx_)pr + τ_θ,_ (15)

I_zz_ r^′^ = (I_xx_ − I_yy_)pq + τ_ψ._ (16)

These equations show how the gyroscopic coupling arises from the inertia asymmetry. For a symmetric quadrotor with I_xx_ = I_yy_, note that two of the coupling terms above simplify to zero.

Each rotor not only generates an upward thrust f_Mi_ but also a reaction torque Mi on the body due to drag on the spinning blade. This motor torque Mi is proportional to the same ω_i_^2^ (with a drag coefficient k_m_) and acts about the rotor’s axis. By design, quadcopters have two pairs of counter-rotating propellers to ensure that in hover the reaction torques cancel out. Their diagonally opposite motors 1 and 3 spin clockwise (CW), while 2 and 4 spin counter-clockwise (CCW), or vice versa.

In hover (all ω_i_ equal), this sum is zero, but by speeding up the CW rotors while slowing the CCW ones (or vice versa), a net yaw torque is produced to rotate the drone [55]. These relations can be summarized in the moment vector:

τ = (τ_φ_, τ_θ_, τ_ψ_) = (l(f_4_ − f_2_), l(f_3_ − f_1_), (M_1_ + M_2_ + M_3_ + M_4_))_._ (17)

In summary, τ_φ_ and τ_θ_ are controlled by differential thrust between opposite rotors, and τ_ψ_ is controlled by the difference in reaction torques between the counter-rotating rotor pairs. By adjusting the individual rotor speeds ω_i_ (the control inputs), a quadcopter can thus modulate T, τ_φ_, τ_θ_, τ_ψ_ to achieve full 6-DOF motion. Each parameter plays a clear role: the total thrust T primarily affects altitude (vertical acceleration), the roll torque τ_φ_ induces banking/rolling motion, the pitch torque τ_θ_ induces nose-up/nose-down pitching and the yaw torque τ_ψ_ controls the heading angle [54,55,56].

#### 3.1.3. Fixed-Wing UAV

A fixed-wing UAV flies by generating lift on its wings to counteract weight and thrust from its engine to overcome drag. The aircraft’s state is typically described by its position, velocity and orientation (often using roll φ, pitch θ and yaw ψ angles similar to the quadcopter). The forces on the airplane include weight W = mg acting downward, lift L generated by the wings (perpendicular to the flight path through the wings), drag D opposing the motion and thrust T from the propeller or jet engine in the forward direction. The basic steady-flight equilibrium is L = W and T = D in straight-and-level flight. To maneuver the airplane, the following control surfaces are used: ailerons (on the wings) control roll φ, the elevator (tail horizontal flap) controls pitch θ (and thus indirectly the lift) and the rudder (tail vertical flap) controls yaw ψ (heading).

When a fixed-wing aircraft executes a level turn (constant altitude turn), it must bank (roll) into the turn. Banking the aircraft by an angle φ tilts the lift force vector, giving it a horizontal component that provides the centripetal force for the turn. Meanwhile, the vertical component of lift is reduced. The rudder is used in coordination to yaw the nose into the turn, preventing the aircraft from skidding or slipping sideways. In a properly coordinated turn, the aircraft’s longitudinal axis is aligned with the flight path, and there is no sideslip (the fuselage points into the turn) [57].

The forces in a coordinated level turn satisfy the following relations. The horizontal component of lift L sin(φ) provides the centripetal force F_c_ required for the turn of radius R at speed V:

L sin(φ) = WV^2^/gR, (18)

where W/g is the mass of the aircraft. The vertical component of lift must balance weight in level flight:

L cos(φ) = W.(19)

From these two equations, one can derive the relationship between bank angle and turn radius. Dividing the first equation by the second eliminates L, giving:

tan(φ) = V^2^/gR, (20)

This is the fundamental equation linking the roll (bank) angle φ to the turning radius R (for a given true airspeed V). A higher φ or higher speed V yields a tighter turn (smaller R). Equivalently, the yaw rate ψ′ is related to φ and V. For a level coordinated turn, the aircraft’s heading change rate ψ′ equals the angular velocity of the turn, ω_turn_ = V/R (assuming negligible wind). Using the relation above, this can be written as:

ψ^′^ = V/R = (g tan(φ))/V, (21)

This formula shows that at a given speed, a larger bank angle yields a higher yaw rate. For example, at 60° bank (tan(φ) = sqrt(3)), the turn radius is R = V^2^/(g × tan(60°)) = V^2^/(g × sqrt(3)) and ψ′ = (g × sqrt(3))/V, implying a very quick turn for moderate V.

To achieve a coordinated turn, the autopilot system must use a combination of aileron, rudder and elevator inputs. Initiating a turn, the ailerons are deflected to roll the aircraft to the desired bank angle φ. As the aircraft banks, the rudder is used to yaw the nose into the turn, aligning the plane with its curved flight path (preventing adverse yaw or slip). The elevator is pulled to increase the wing’s angle of attack, raising the lift L so that L × cos(φ) continues to equal W despite the tilt in lift. Thrust may also be increased to counteract the extra drag that results from increased lift in the turn. In steady coordinated turning flight, the aircraft will have a constant bank angle and turn rate, with the forces balanced as per the above equations. The roll angle φ thus primarily controls the turn tightness (radius R or rate ψ′) for a given speed, while the pitch controls altitude (via L) and the rudder ensures the turn is coordinated (maintaining a zero sideslip angle). The relationship tan(φ) = V^2^/gR is a key outcome: it quantitatively links the geometric turn radius to the bank angle and is analogous to the requirement that birds and airplanes must bank into a turn to generate centripetal force. This derivation and these principles are supported by standard flight dynamics analyses [58].

#### 3.1.4. Bird Flight Specifics

In soaring mode, the bird’s silhouette resembles a glider, with a wide base near the body, a gradual taper towards the tip and a slight twist (washout) that reduces the risk of flow disruption at the tips. The primary feathers form fingers at the wing tips, creating an effect similar to winglets that reduces inductive drag [59]. In addition, the bird can partially fold its wings or unfold its tail, changing the effective area and aerodynamic properties. This mechanism works similarly to the use of flaps or air brakes in aircraft [60].

In flight, a bird can be considered as a rigid body with fixed wings. The basic equations of motion—the balance of forces in straight-line flight, the relationship between bank and turn radius and the change of course during a coordinated turn—are similar to the fixed-wing model. At the same time, in real flight, small fluctuations in yaw and roll are observed due to micro-corrections by the feathers and tail, which gives the silhouette instability and distinguishes it from fixed wing.

For example, if a bird moves at a constant speed, the Kalman filter can predict its new position even if it momentarily disappears behind an obstacle, and when the object reappears, the measurement helps to refine the prediction.

Mathematical differences from fixed wing:

- The area S and the coefficients CL, CD vary depending on the position of the feather or tail, while in fixed wing they are constant [59];
- A bird can move from a stable to a less stable mode by changing the position of its wings or tail. This makes the neutral point movable, unlike in fixed wing where it is structurally fixed [60].

### 3.2. Flight Dynamics Modeling

In this work, we model three representative bird types in order to capture the diversity of avian flight behaviors and to avoid relying on a single “generic bird” trajectory. Specifically, we simulate a pigeon, a gull and a peregrine falcon. These species were selected because they differ markedly in typical cruising speed, agility and maneuver intensity, which directly affects the temporal structure of their trajectories. In the model, each bird type uses the same unified kinematic formulation introduced below, but with species-specific parameter ranges (e.g., preferred speed, maximum bank angle and noise and perturbation levels), allowing the generated synthetic trajectories to reflect distinct flight signatures while remaining comparable within a common mathematical framework.

We define motion in a fixed Earth reference frame:

F_E_ = {x = [x, y, z]^T^}, (22)

At time t: x(t) ∈ R^3^ is position of the agent (bird or drone); v(t) = x’(t) is velocity in the Earth frame; u(t) = v(t)/‖v(t)‖ is unit forward direction; s(t) = ‖v(t)‖ is speed magnitude.

The agent is subject to deterministic and stochastic accelerations determined by control, aerodynamics and environment.

The bird’s equations are a kinematic model derived from turn–bank relations, with lateral acceleration proportional to the tangent of the bank angle:

x, u, s, ϕ,(23)

where x is the position; u is the flight direction (unit vector); s is the airspeed magnitude; ϕ is the bank angle (controls turning curvature).

The model of the bird is a point mass moving with orientation vector u(t) (the direction of forward flight), scalar speed s(t) and position x(t).

#### 3.2.1. Translational Kinematics

The object’s translational motion is modeled in the Earth fixed frame by combining its airspeed along the forward direction with the local wind velocity at its position. This yields the following relationship for the position derivative:

x^′^ = s × u + w(x), (24)

where x = [x, y, z]^T^ is the 3D position in the Earth reference frame; s is the scalar airspeed of the bird (relative to air); u is the unit heading vector, ‖u‖ = 1; w(x) is the local wind velocity field, which adds drift relative to the ground.

This means the bird’s ground relative velocity equals the sum of:

- Its self-propulsion s u;
- The environmental wind at its position.

#### 3.2.2. Direction Kinematics

The forward direction is represented by the unit vector u(t) and is assumed to rotate with angular velocity ω(t). Its kinematic evolution is therefore described by:

u^′^ = ω × u, ‖u‖ = 1, (25)

where ω is the angular velocity vector describing how the bird’s forward direction rotates in space.

Because u is always a unit vector, its derivative must be orthogonal to itself. The cross-product form guarantees that u·u’ = 0. The direction of ω is along the axis of rotation (perpendicular to the flight plane), and its magnitude is the turn rate (radians per second).

#### 3.2.3. Speed Dynamics

This is a first-order relaxation model toward a preferred airspeed, with environmental coupling and random fluctuations:

s^′^ = k_s_ (s* + β_tw_ (u⋅w) − s) + σ_s_ξ_s_(t), (26)

where s* is the nominal or species-specific cruise speed (e.g., 12 m/s for pigeon); k_s_ is the rate constant controlling how quickly speed adapts; β_tw_ is the sensitivity to tailwind—if flying with wind (u·w > 0), speed increases slightly, against wind, decreases; σ_s_ is the amplitude of random variation (e.g., turbulence, small energy fluctuations); ξ_s_(t) is the zero-mean white Gaussian noise.

#### 3.2.4. Bank Angle Dynamics

ϕ^′^ = k_ϕ_ (ϕ* − ϕ) + σ_ϕ_ ξ_ϕ_(t), (27)

where ϕ is the current bank angle (roll), which determines lateral acceleration; ϕ* is the desired bank computed from guidance behavior (goal attraction, wind compensation, obstacle avoidance); k_ϕ_ is the roll control rate (how fast the bird changes bank); σ_ϕ_ is the scalar constant (noise strength, amplitude); ξ_ϕ_(t) is the random white noise process.

#### 3.2.5. Coupling via Lateral Acceleration

The angular velocity ω is determined by the lateral acceleration the bird experiences:

ω = (u × a_L_)/s, (28)

and the lateral acceleration magnitude is limited by lift:

‖a_L_‖ = g tan(ϕ), (29)

where g = 9.81 m/s^2^.

This means that small ϕ is gentle turning (large radius); large ϕ is sharp turning (small radius).

In the proposed bird flight simulator, stochastic parameters are used to reproduce the natural, non-deterministic variability of biological motion while preserving kinematic continuity and physical plausibility. Speed noise (σ_s_) introduces realistic short-term fluctuations around the nominal cruise speed, reflecting intermittent flapping, effort changes and energy management, whereas turning-related perturbations (σ_a_, σ_u_, σ_φ_) add small random variations in lateral acceleration, heading and bank angle dynamics, preventing unrealistically smooth trajectories and producing curvature irregularities consistent with wing asymmetries, neuromuscular control noise and gust responses. In addition, environmental stochasticity is modeled via a temporally correlated wind gust process, which creates sustained disturbances that lead to drift correction behavior typical for real flight rather than frame-to-frame jitter. Importantly, all stochastic terms are constrained (bounded by limits such as maximum bank angle and gravitational acceleration and applied in a physically consistent direction), so increased variability yields diverse trajectories without violating biomechanical feasibility.

Parameters that are used in calculations can be checked in Table 1.

### 3.3. Neural Network Model

The classifier is formulated as a binary decision (“bird-like” vs. “drone-like”), reflecting the first-stage requirement of a hierarchical surveillance pipeline. Here, “drone-like” denotes artificial UAV motion to be further processed by downstream tracking/response modules, whereas “bird-like” denotes biological motion that can be filtered out to reduce false alarms.

The classification model can be a bidirectional long short-term memory (LSTM) network. This type of model is well-suited for sequential data because it can learn temporal dependencies between frames. A bidirectional LSTM processes the sequence both forward and backward, enabling it to consider both past and future context when making predictions for each frame.

The model architecture has properties as shown in Table 2.

Each sequence produces an output of the same length as the input—one probability per frame. The average of these probabilities is used as the overall prediction for the entire trajectory.

The loss is computed using binary cross-entropy (BCE) between the predicted probabilities and the true labels. Because sequences are padded to equal length for batch processing, a mask is applied to ensure that padding did not affect the training loss.

The masked BCE loss is defined as:

Loss = (Σ_i_ mask_i_·BCE(p_i_, y_i_))/(Σ_i_ mask_i_), (30)

This ensures that only valid frames contribute to the loss. The model is trained using the Adam optimizer, with a learning rate of 1 × 10^−3^. Training is performed for 35 epochs, and the model with the highest AUC score on the validation set is saved as the best performing version.

### 3.4. Training Strategy

**Figure 3:**
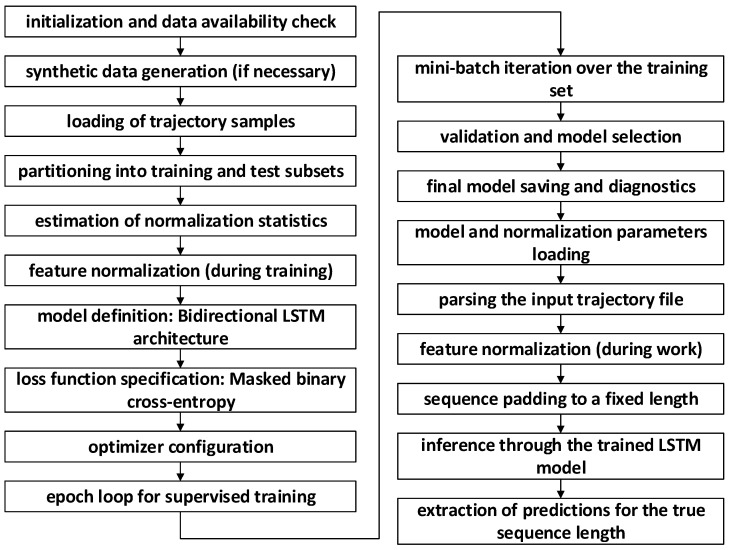
Steps of drone-bird classifier training.

An algorithm to train the drone–bird classifier has steps shown in Figure 3.

The algorithm first verifies whether the trajectory dataset folder contains any samples. This check ensures that subsequent steps operate on a non-empty collection of trajectory sequences.

If the dataset folder is found to be empty, the procedure to generate synthetic dataset is invoked. This routine programmatically generates labeled trajectory samples that mimic the motion characteristics of birds and drones. The resulting synthetic data are stored in the dataset folder in a consistent JSON format, ensuring that the subsequent loading stage can proceed without manual data collection.

All available JSON samples in the dataset folder are loaded into memory. Each JSON file is parsed into numerical tensors representing the sequence of 3D positions (x, y, z) over time, together with a corresponding class label y ∈ {0, 1}, where 0 denotes “bird-like” and 1 denotes “drone-like”. The sequence lengths may vary across samples; this variability is handled later via masking in the loss function.

The full set of loaded trajectories is partitioned into two disjoint subsets: train set and test set. The training subset is used for parameter optimization of the LSTM model, while the test subset is reserved exclusively for performance evaluation (validation AUC), thereby preventing information leakage. The splitting strategy may be random or stratified, but it must preserve the class distribution as much as possible to avoid bias.

For all trajectories in the training subset, the algorithm computes the empirical mean vector μ and standard deviation vector σ across the three input features (x, y, z). These statistics are computed solely on the training data to avoid contaminating the model with information from the held-out test set.

Each trajectory sample, in both the train set and test set, is normalized feature-wise using the affine transformation:

X_norm_ = (X − μ)/σ, (31)

where the subtraction and division are performed per feature dimension.

This standardization centers the data around zero and scales it to unit variance, which is known to improve the stability and convergence properties of gradient-based training for recurrent neural networks.

The classifier is instantiated as a bidirectional long short-term memory (LSTM) network with the following configuration:

- Input dimension: 3 (corresponding to the three input channels (x, y, z) per time step);
- Hidden dimension: 64 (indicating that each LSTM direction maintains a 64-dimensional hidden state);
- Layers: two (implying a two-layer stacked LSTM, which allows the model to learn hierarchical temporal representations).

Bidirectionality enables the model to exploit both past and future context within each trajectory sequence, which is particularly beneficial when discriminating motion patterns that may depend on global shape rather than purely causal dynamics.

The LSTM outputs a sequence of hidden states that is then aggregated (e.g., via pooling or final time step selection) into logits representing the predicted probability of the “drone-like” class.

The training objective is defined, a variant of binary cross-entropy adapted for variable-length sequences. Each batch contains sequences that may have been padded to a common maximum length; the associated mask tensor indicates which time steps correspond to valid data and which correspond to padding. The masked BCE loss computes the element-wise cross-entropy between predicted logits and ground truth labels only on valid positions, ignoring padded elements in both the forward and backward passes. This design enables efficient mini-batch training while maintaining a correct loss signal for unequal sequence lengths.

Optimization is performed using the Adam optimizer with a learning rate of 0.001, updating all trainable parameters of the LSTM model. Adam combines adaptive learning rate scaling with momentum-like estimates of first and second-order moments of the gradients, which typically yields robust convergence across a range of deep learning tasks. The optimizer is configured to update all trainable parameters of the LSTM model.

The algorithm performs an outer loop over epoch from 1 to 35 (inclusive), corresponding to a fixed training schedule of 35 epochs. At the beginning of each epoch, the model is set to training mode, which enables certain behaviors such as dropout (if present) and ensures that any internal layers behave appropriately for training rather than evaluation. A scalar accumulator total loss is reset to zero to record the sum of mini-batch losses over the epoch.

The training data are accessed through a train loader iterator, which yields batches of trajectories. For each batch, the algorithm unpacks three tensors:

- X: a batch of normalized trajectory sequences;
- y: the corresponding batch of binary labels (0 for bird, 1 for drone);
- mask: a binary mask identifying valid time steps in each sequence.

The model computes the output, where we get unnormalized scores for class membership at the sequence level. The loss value for the batch is then evaluated, which applies the masked binary cross-entropy objective. Before performing backpropagation, accumulated gradients are cleared by invoking the optimizer’s special function. The gradient of the loss with respect to all model parameters is calculated. The optimizer then updates the parameters using the special function, applying the Adam update rule. The total loss accumulator is updated by adding the current batch loss, optionally scaled by batch size, to enable later reporting or plotting of training loss trends.

After all training batches for the current epoch have been processed, the model’s performance is evaluated on the held-out test set. The primary validation metric is the area under the ROC curve (AUC), which measures the model’s ability to discriminate between bird and drone trajectories across all classification thresholds. The validation AUC is computed by running the model in evaluation mode over the test set and aggregating predicted probabilities and true labels. If the current epoch’s validation AUC exceeds the best previously recorded value, the current model parameters are saved as the “best” model. This implements an early model selection strategy based on validation performance, mitigating overfitting.

After completion of all epochs, the algorithm saves the final trained model parameters to disk, ensuring that the classifier can be reused for inference or further fine-tuning. In addition, the history of training loss values (and optional validation AUC per epoch) is saved and used to generate a training loss plot. This plot provides a visual diagnostic of convergence behavior, enabling inspection for signs of underfitting, overfitting or learning rate mismatch. An algorithm makes a prediction on a new trajectory.

The prediction pipeline begins by loading the previously trained LSTM classifier from the disk. Along with the model, the algorithm loads the feature normalization statistics—specifically, the mean vector μ and standard deviation vector σ—that were computed from the described previous algorithm. Using the same normalization parameters ensures consistency between training and inference, preventing distributional mismatch and preserving classifier reliability.

The algorithm reads the JSON input file supplied before running. From this file, it extracts the movement history field, which contains the sequence of 3D positions representing the new, unlabeled trajectory. The trajectory is converted into a numerical tensor of shape (T,3), where T denotes the actual sequence length. Any auxiliary metadata in the JSON file are ignored at this stage, as prediction is based exclusively on positional information.

Each time step of the trajectory is normalized using the affine transform:

X_norm_ = (X − μ)/σ, (32)

where μ and σ are applied feature-wise.

This transformation ensures that the new trajectory is brought into the same feature space as the data used to train the LSTM, thereby guaranteeing numerical compatibility and stable model behavior.

The trained LSTM expects batches of sequences with a uniform maximum length, typically set to a maximum length equaling 100. If the input trajectory length T < 100, the algorithm pads the normalized sequence with zeros until the required maximum length is reached. A corresponding binary mask is constructed internally to distinguish valid positions from padded time steps during the forward pass, although this mask may not be explicitly returned to the user. If T > 100, the sequence may be truncated or handled according to the implementation detail, but by design the padding step ensures that the LSTM receives a tensor of consistent dimensionality.

The padded, normalized trajectory is passed through the bidirectional LSTM classifier. The LSTM processes the sequence to produce logits, i.e., unnormalized outputs corresponding to the probability that each frame originates from a drone rather than a bird. These logits are transformed via the logistic sigmoid function producing probabilities in the interval [0, 1] for each time step. Since bidirectional LSTMs incorporate both past and future context, each probability reflects a global assessment of the movement characteristics at that frame.

The model produces a sequence of maximum lengths, but only the first T entries correspond to actual observed data. The algorithm removes predictions associated with padded time steps, which yields a one-dimensional array of per-frame drone probabilities for the original, unpadded trajectory. These values constitute the final output, enabling downstream systems to interpret the likelihood of drone-like behavior at each observed time step.

Having defined the simulation environment, trajectory encoding and LSTM-based classifier, we now define how these components are generated in our experiments. Section 4 (Experimental Setup) details the software implementation, parameter settings and synthetic data generation protocol used to generate the training and test sets. This ensures that the results obtained are reproducible and that differences in performance can be explained by controlled experimental factors.

## 4. Experimental Setup

### 4.1. Used Libraries

For the experimental setup, we used Python (3.13.5). The following libraries were used: json (2.0.9), numpy (2.3.3), torch (2.8.0), sklearn (1.7.2), matplotlib (3.10.6), opencv-python (4.12.0.88), pandas (2.3.2) and pillow (11.3.0).

**Figure 4:**
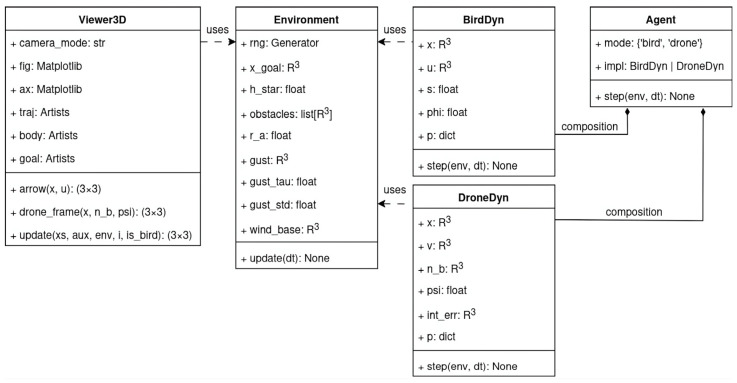
Class diagram.

Inside the code there are five different classes: Viewer3D, Environment, BirdDyn, DroneDyn and Agent. You can see the members for every class in Figure 4.

### 4.2. Generation of Synthetic Trajectories for Flying Objects

The code implements a unified stochastic simulation pipeline for modeling three-dimensional trajectories of flying birds and a quadrotor-type drone. It integrates dynamical models, an environmental wind field, numerical integration, data export and 3D visualization into a single pipeline, controlled through a simple interface.

The environment class encapsulates external conditions:

- A fixed 3D waypoint that both birds and drone tend to approach;
- A preferred flight altitude used by the bird for altitude regulation;
- A list of obstacle positions, used for repulsive avoidance;
- A constant background wind vector; parameters of a stochastic gust model.

The environment maintains a 3D gust vector that evolves according to an Ornstein–Uhlenbeck (OU) process. This process generates temporally correlated random wind perturbations with finite variance rather than uncorrelated white noise. The responsible method then returns the total wind as the sum of the base component, the OU gust term and a simple “thermal” vertical component that depends on the horizontal distance from a fixed center. This yields a three-dimensional, time-varying wind field that influences both birds and drone.

The BirdDyn class implements a kinematic stochastic model of a fixed-wing bird:

- State variables: position, unit forward direction, speed and bank angle;
- Special parameters encode species-specific behavior (cruise speed, maneuverability, noise intensities, etc.).

At each time step dt, computes several behavioral acceleration components:

- Lateral attraction toward the goal position, projected perpendicular to the current heading;
- Altitude control, driving the bird toward the preferred flight height;
- Cross-wind compensation, turning into the wind to reduce drift;
- Repulsive acceleration away from obstacles, decaying with distance;
- Lateral random acceleration, drawn from a Gaussian distribution and projected perpendicular to the unit vector.

The sum converts into a desired bank angle by relating lateral acceleration magnitude to gravitational acceleration via an arc tangent to the gravitation, with saturation at a species-specific maximum. The actual bank angle follows via a first-order stochastic differential equation, which introduces both finite responsiveness and noise. The resulting lateral acceleration is then capped to respect physical limits.

The orientation vector is updated using Rodrigues rotations about an axis defined by the cross product, which implements curvature in a geometrically consistent way. Additional heading noise is then injected orthogonally into the unit vector. Speed s is updated via relaxation toward a reference speed s* modulated by wind alignment and perturbed by Gaussian noise. Finally, the position x is advanced using the sum of airspeed and the local wind w.

Species-specific parameters are constants that yield distinct parameter sets for pigeons, gulls and peregrine falcons, thereby inducing qualitatively different trajectory styles (e.g., slower, more meandering gull flight versus fast, agile peregrine motion).

The DroneDyn class models a simplified quadrotor as a point mass with:

- Position x and velocity v;
- Body thrust direction (a unit vector), initially vertical;
- Yaw angle ψ;
- An integral error term for position control.

At each step, the drone computes:

1. Position error relative to x goal and altitude error relative to h*;
2. A PID-style desired acceleration combining proportional, derivative and integral terms for position, plus altitude and wind compensation contributions;
3. An obstacle avoidance term, analogous to that used for birds.
4. An additive Gaussian noise term on acceleration, modeling imperfect control.

Acceleration is then saturated separately in the horizontal and vertical components. A desired thrust direction is obtained by adding gravity and normalizing. The actual thrust direction is rotated toward the desired thrust direction with a finite maximum tilt rate and capped by a maximal tilt angle. This enforces realistic rotational constraints for the drone.

The required thrust magnitude is computed by projecting the desired force along actual thrust direction and saturating it at a specified maximum. Yaw is controlled to approximately align either with the velocity vector or with the goal direction, again with saturation and small noise. Finally, the translational dynamics are updated with a simple ground constraint ensuring that the drone does not pass below (z = 0).

### 4.3. Trajectory Simulation

To evaluate the proposed trajectory-based drone–bird classifier under controlled conditions, we generated a synthetic dataset of three-dimensional movement trajectories. The dataset was produced by a custom stochastic simulator that models the kinematics of flying birds and a quadrotor-type drone within a shared 3D environment.

**Figure 5:**
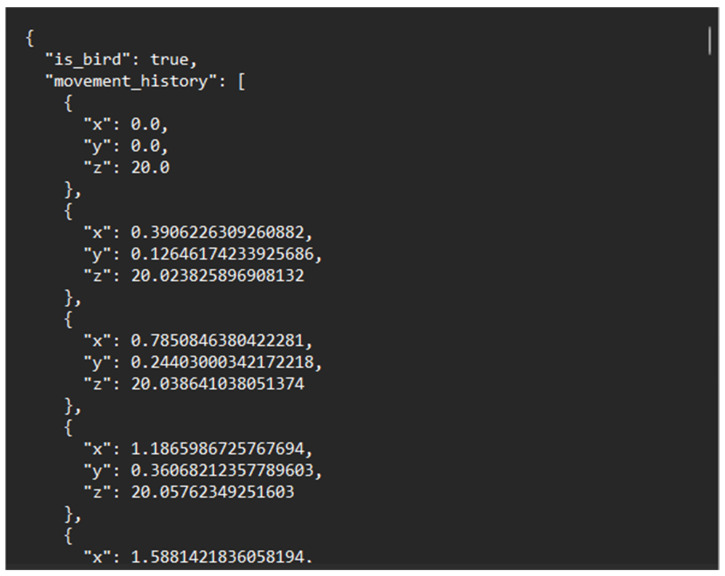
Example of pigeon trajectory in JSON file.

Each simulation instance produces a single trajectory with multiple points that is stored in a JSON file. The JSON structure contains a Boolean flag indicating whether the agent is a bird or a drone and an ordered list of positions as shown in Figure 5.

The special Viewer3D class wraps Matplotlib’s 3D plotting tools to provide an animated visualization of the trajectory. The constructor sets up:

- A 3D axes object with labeled axes and fixed limits;
- A ground surface (greenish plane at z = 0) and a sky surface (bluish plane at high altitude) to give spatial context;
- Line artists for the trajectory (self.traj) and the current body representation (self.body), and a scatter point for the goal.

For birds, a special function constructs a small triangular “arrow” aligned with the heading vector. For the drone, a responsible function constructs a simple shape aligned with the thrust direction and yaw. The update method updates these artists for a given time index and optionally adjusts axis limits to keep the agent approximately centered in a “follow” camera mode.

**Figure 6:**
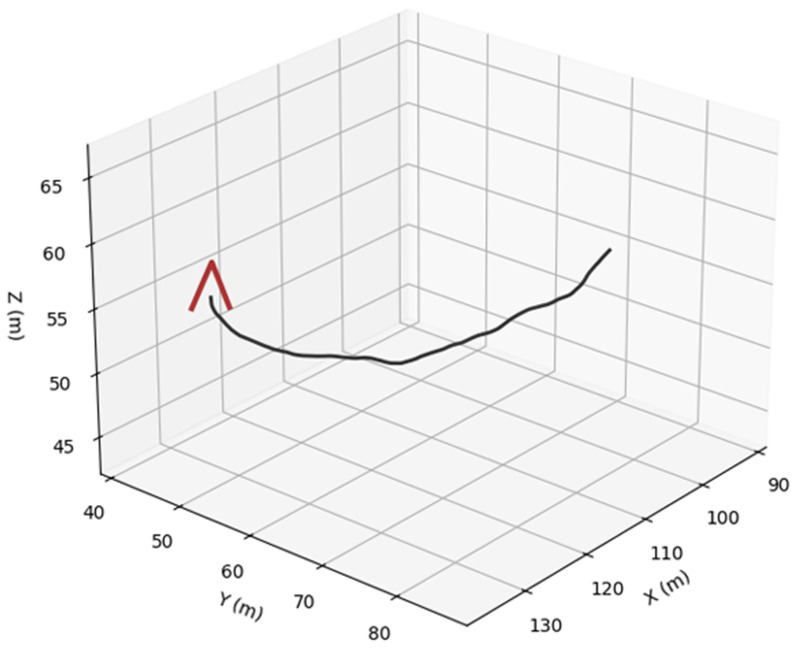
Pigeon simulation.

**Figure 7:**
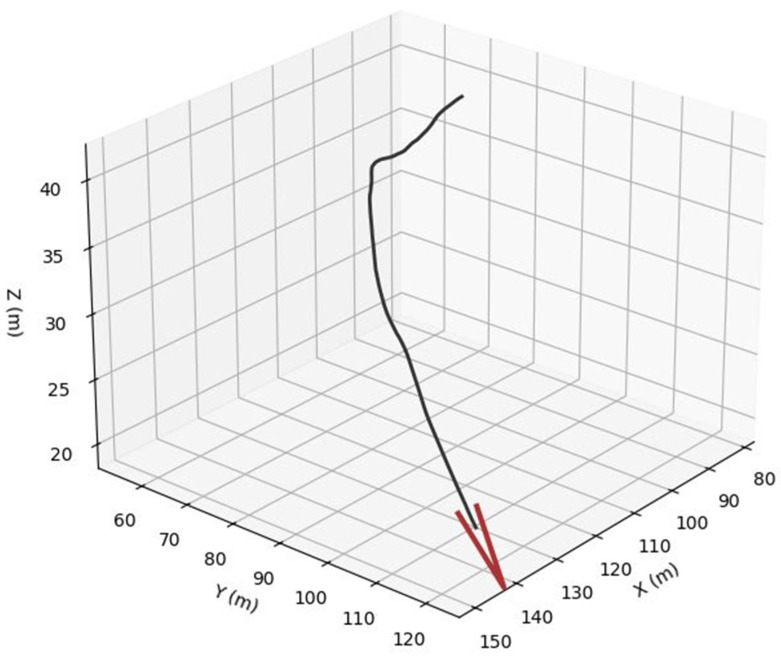
Gull simulation.

**Figure 8:**
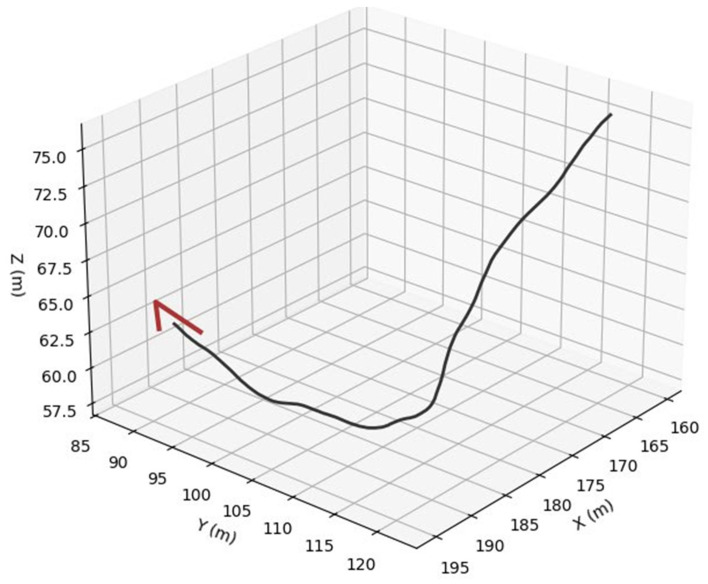
Peregrine simulation.

**Figure 9:**
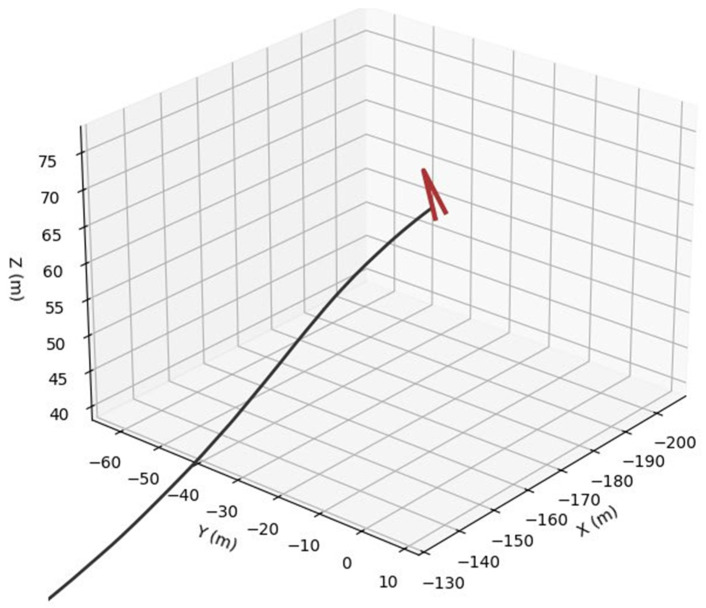
Drone simulation.

For bird trajectories, three species presets are used: pigeon (Figure 6), gull (Figure 7) and peregrine (Figure 8). They differ in nominal cruise speed, bank angle limits and sensitivity to wind, altitude and goal attraction. For drone trajectories (Figure 9), a simplified quadrotor model is employed with PID-style position control, wind compensation, obstacle avoidance and additive control noise. In all cases the environment provides a time-varying wind field with Ornstein–Uhlenbeck gusts and fixed obstacles, and all simulations are run in discrete time with step size dt = 0.03 s over a chosen horizon T (e.g., 100 s).

The data generation pipeline is integrated with the training code as follows. The special function runs the simulator multiple times with random seeds and parameter draws, producing a balanced set of bird and drone trajectories. The resulting JSON files form the raw dataset used for training and evaluation.

All JSON files in the dataset directory are parsed and converted into numerical tensors. For each trajectory, the 3D coordinates are stacked into a sequence X ∈ RT × 3 with features (x, y, z) per frame, and a binary label y ∈ {0, 1} is assigned, with 0 corresponding to “bird-like” and 1 to “drone-like”.

**Figure 10:**
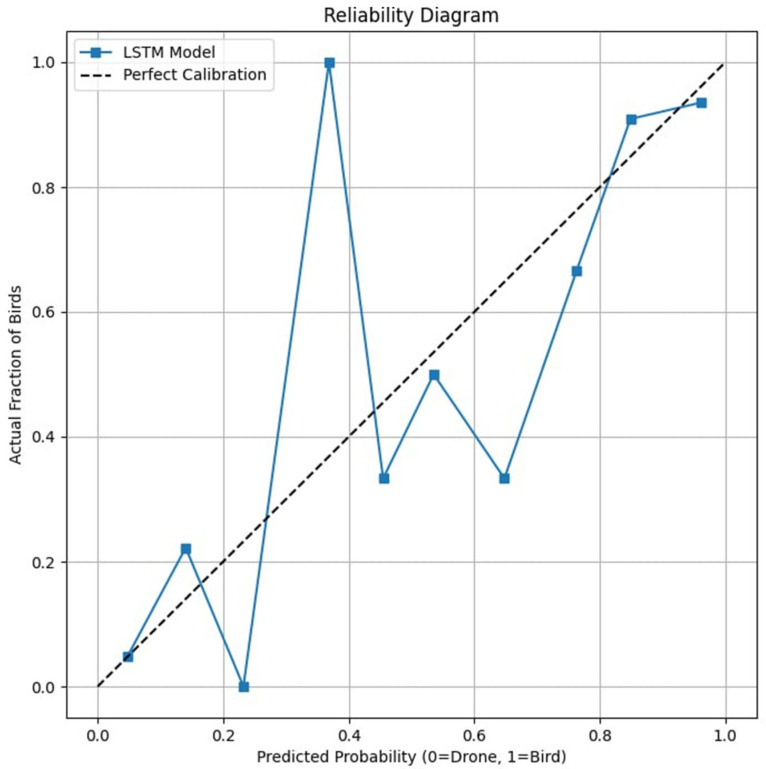
The reliability diagram of LSTM network.

The classifier is implemented as a bidirectional long short-term memory (LSTM) network. At each time step, the network receives a three-dimensional input vector (normalized (x, y, z)). The model uses an input dimensionality of 3, a hidden state size of 64 units per direction, and two stacked LSTM layers, yielding a deep recurrent architecture capable of capturing temporal dependencies across the full trajectory. Bidirectionality enables the network to exploit both past and future context along the sequence, which is appropriate in this setting as the entire trajectory is available at inference time. The reliability of LSTM network is shown in Figure 10.

Given the experimental protocol and parameter settings defined in Section 4, we now evaluate the proposed pipeline from modeling to classification. Section 5 (“Results”) reports the quantitative performance of the trajectory-based LSTM classifier and analyzes how motion-derived cues support the discrimination of drones and birds under long-range appearance constraints. We also summarize key observations from synthetic data validation to confirm the robustness of the generated trajectories.

## 5. Results

### 5.1. LSTM Model Training

**Figure 11:**
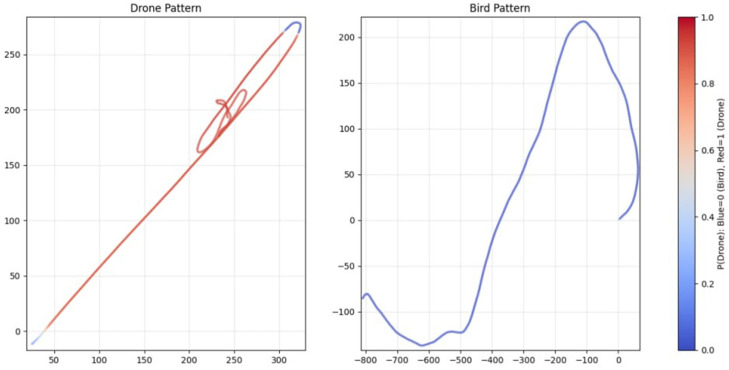
Trajectory movement patterns for bird and drone.

To assess the realism and internal consistency of the simulated bird and drone trajectories, we conducted a validation study focused on the physical plausibility, behavioral diversity and statistical properties of the generated motion patterns shown in Figure 11. Because no real trajectory dataset was available for quantitative comparison, we evaluated the synthetic trajectories against (i) known biomechanical and aeronautical constraints reported in the literature and (ii) qualitative expectations of flight dynamics.

#### 5.1.1. Physical Plausibility of Motion

The bird model is based on lateral acceleration constraints derived from bank angles, the gravitational constant and biomechanically plausible speed limits. We verified that all simulated trajectories obeyed these constraints:

- No lateral acceleration exceeded g tan(ϕ_max_);
- Speeds remained within the expected species-specific ranges;
- Altitude changes were smooth and continuous;
- Trajectories avoided kinematic discontinuities.

#### 5.1.2. Behavior Consistency with Species Flight Dynamics

Simulated species exhibited characteristic motion patterns commonly described in avian biology:

- Pigeons showed moderate speed and relatively direct goal-oriented flight;
- Gulls displayed smoother, more drifting motion with pronounced wind interaction;
- Peregrine trajectories exhibited higher speeds and sharper curvature.

These qualitative patterns matched established observations reported in the biomechanics literature, providing evidence that the parameterization effectively captures inter-species variability.

#### 5.1.3. Statistical Analysis of Trajectory Properties

For each synthetic dataset, we computed descriptive statistics such as:

- Distribution of instantaneous speed;
- Distribution of turning curvature;
- Histogram of altitude changes;
- Autocorrelation of velocity vectors;
- Variance introduced by the wind gust model.

All observed distributions were smooth, non-degenerate and consistent with the expected behavior of stochastic flight models. Trajectories exhibited natural variability due to the Ornstein–Uhlenbeck wind field and random acceleration perturbations.

#### 5.1.4. Diversity and Non-Determinism

Using different random seeds yielded trajectories with significantly different spatial paths, curvature profiles and timing characteristics. This confirms that the simulator does not produce artificially repetitive motion and is capable of generating a diverse dataset suitable for training machine-learning models.

#### 5.1.5. Classification Performance

**Figure 12:**
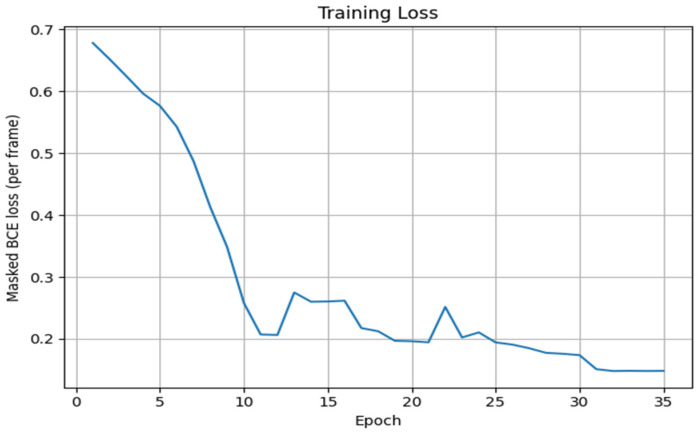
Training loss over epochs.

Training was conducted on a CPU (12th Gen Intel(R) Core(TM) i5-12600KF) for demonstration purposes, though the code supports GPU (NVIDIA GeForce RTX 3070ti) acceleration. The model converged steadily, as shown in Figure 12.

This plot shows the masked binary cross-entropy loss computed per frame during training. The loss decreases rapidly during the first 10 epochs and stabilizes around 0.15 after 30 epochs, indicating that the model has effectively learned to distinguish the classes. The steady decline and stabilization of the loss function suggest that the model generalized well without severe overfitting.

**Figure 13:**
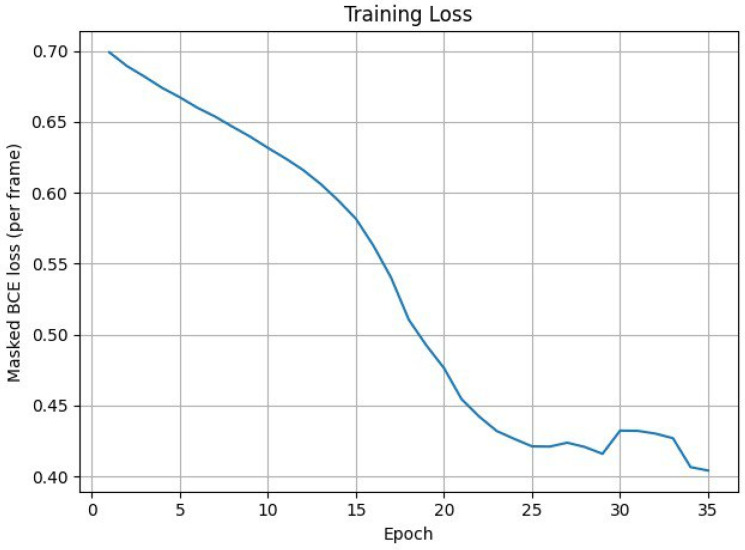
Training loss over epochs for gated recurrent unit model.

To ensure the model is chosen correctly for this task, a different model was tested. For this purpose, a gated recurrent unit architecture was selected and trained on the same dataset (Figure 13).

The plot also shows steady decline and stabilization of loss function, with some differences. In comparison with the LSTM model, usage of a gated recurrent unit in training slows down the training process and the loss function stabilization happens at a 0.4 rate in comparison with the LSTM model where this happens around 0.15. Based on this comparison it is possible to state that the LSTM model is preferred in this classification task before the gated recurrent unit.

**Figure 14:**
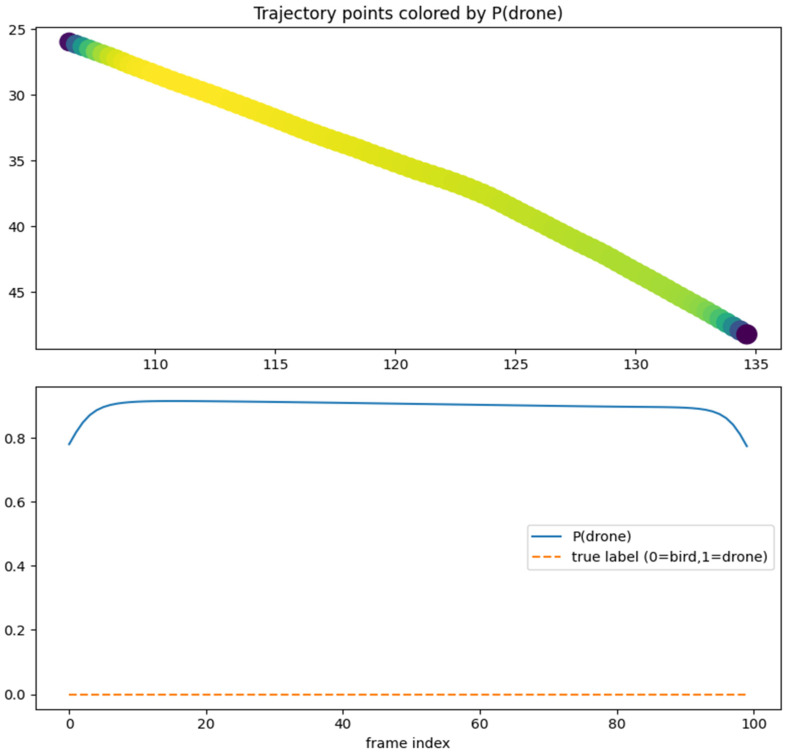
Example prediction visualization.

After training, the model was tested on previously unseen sequences. Each test sample was fed into the network, which output the probability P(drone) for each frame. An example of a correctly classified drone trajectory is shown in Figure 14.

The upper plot displays the trajectory of a moving object, with color intensity representing the predicted probability of being a drone. The lower plot shows the probability curve P(drone) across frames compared with the true label. The model consistently assigns high confidence to drone frames (around 0.9), demonstrating reliable temporal predictions.

The visualization helps to verify that the model not only produces correct overall classifications but also maintains stable per-frame predictions across the entire sequence.

The demonstration stage, which loads the PyTorch v.2.8.0 trained model, computes per-frame drone probabilities for a new trajectory. The user provides a path to a JSON file as an argument. The results are shown in Table 3.

The demonstration confirms that the model can accurately classify new, unseen motion sequences by analyzing only their spatial and temporal behavior, without visual information. This approach could be particularly useful in scenarios where video resolution is low or where objects are too distant for clear identification.

### 5.2. Comprehensive Tracking Pipeline

A complex tracking system with a multi-camera and a multi-level approach allows to detect an object that is barely noticeable at first (from 5 pixels), and then gradually becomes larger. In this paper, we present the necessary technologies that allow such analysis. To sum up, the entire technology forms a coherent workflow consisting of stages presented below.

#### 5.2.1. Working with a Very Small Object (5–10 Pixels)

At a large distance, the object occupies only a few pixels, so using classic CNNs or transformers is inefficient here as they need enough information in the image to learn to distinguish shapes. In this case, simpler algorithms that work with blobs and brightness features are more appropriate.

LoG (Laplacian of Gaussian), DoG (difference of Gaussians), DoH (determinant of Hessian) and MSER can be used as detection methods.

**Figure 15:**
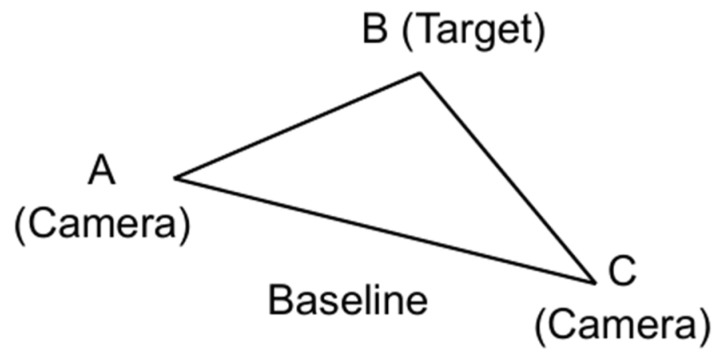
Triangulation work principle.

Since one camera only provides 2D coordinates, we use multiple cameras. Based on the positions of the blob in 2D images, we can use triangulation to reconstruct the approximate position in 3D as shown in Figure 15. This allows us to work directly with spatial coordinates (*X*, *Y*, *Z*).

At this stage, the Kalman filter in 3D (or particle filter) is important. It helps smooth the trajectory and makes the system resistant to noise (after all, a small object can easily be confused with noise or pixels in the background).

#### 5.2.2. Association and Tracking Between Frames

When an object is identified in multiple cameras, the task is to understand that this point in frame *t* corresponds to the same point in frame *t* + 1. For data association, nearest neighbor, Hungarian algorithm, JPDA or MHT can be used.

Tracking between frames is based on a prediction (Kalman or particle filter) and association with new detections. This allows the object ID to be maintained even when it disappears from view for several frames.

#### 5.2.3. Moving to More Complex Methods (The Object Becomes Larger)

When an object approaches or flies closer to the camera, its projection already takes up tens or hundreds of pixels. Now you can use neural networks for detection.

CNN detectors (YOLO, Faster R-CNN, RetinaNet, Deep SORT) and tracking transformers (TrackFormer, TransTrack) can be used here [61].

Here, a classification aspect is already possible as a neural network can determine whether the object in the frame is a drone or a bird. This is critically important, because at a long distance along the trajectory they are similar, but as the size increases, information about the shape and structure appears.

#### 5.2.4. Integration of All Subsystems

**Figure 16:**
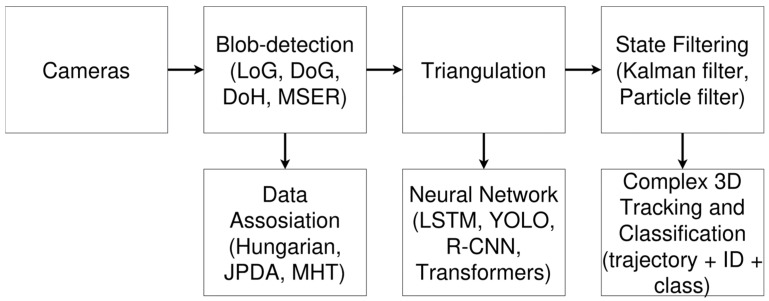
Classification pipeline.

As a result, we obtain a complex pipeline (Figure 16): At a long distance, when the object is small, simple blob detectors and triangulation from several cameras work. Here, the geometry of the cameras and state filtering play a key role.

When the object enters the zone of better visibility, a more powerful system is connected to the work—CNN or transformer—that is able to identify the object and track it. At the level of data association, methods like Hungarian or Deep SORT always work, as they connect trajectories in time and maintain the correct ID. The output is a continuous 3D trajectory of the object in space with its classification (bird or drone).

### 5.3. Calculated Metrics

**Figure 17:**
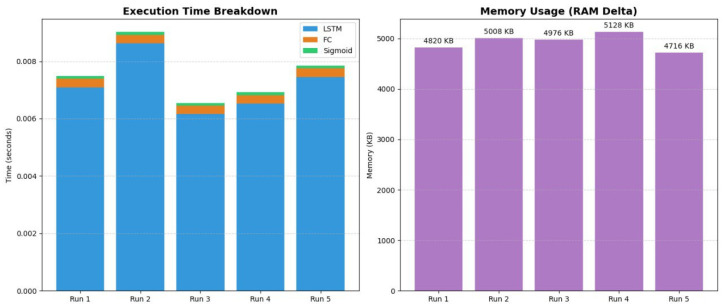
Classification metrics.

For the classification job two metrics were calculated: inference time and memory occupancy. The task was performed on five sets of new data, each containing 100 steps, and the results are shown in Figure 17.

As it is shown in the diagram, the average time for the tasks does not exceed 7 milliseconds. Also calculations of the linear layer (FC) and the sigmoid take the same amount of time per run, as both of these methods contain one formula that takes constant time for calculation. Average memory usage does not exceed 5 megabytes.

## 6. Discussion

The results obtained in this study demonstrate that simulated trajectories can serve as an effective proxy for training early-stage deep learning models aimed at distinguishing drone and bird flight behavior. Despite the absence of real-world trajectory data, the proposed simulation pipeline produced diverse, physically plausible flight paths that capture essential differences between biological and mechanical flight. The neural network trained on these synthetic sequences exhibited stable convergence and a clear ability to discriminate between the two classes, suggesting that the temporal dynamics encoded in the synthetic data are sufficiently expressive for machine-learning purposes.

Nevertheless, several limitations must be acknowledged. First, although the simulation incorporates biologically informed parameters (e.g., bank-angle constraints, species-specific speed ranges, wind compensation), it cannot fully reproduce the complexity and variability of real avian motion. Similarly, the drone model simplifies aerodynamic and control-system dynamics, omitting noise sources such as sensor uncertainty or communication delays. These simplifications may introduce discrepancies between simulated and real trajectories that could affect generalization performance once real data become available. Second, because no real dataset was available for validation, the realism of the simulated trajectories was assessed only in terms of biomechanical plausibility and consistency with the literature, rather than direct empirical comparison.

Future work should focus on collecting and integrating real-world flight recordings, using radar, optical tracking or telemetry data. Such datasets would allow a more rigorous evaluation of simulation fidelity and would enable fine-tuning of the classifier for practical deployment. Additionally, incorporating more complex environmental factors such as obstacle-rich terrains, stronger turbulence models or multi-agent interactions could further improve the realism and diversity of synthetic trajectories.

## 7. Conclusions

This work introduces a complete pipeline for generating synthetic 3D flight trajectories of birds and drones, and for training a recurrent neural classifier capable of distinguishing between the two classes based on their motion patterns. The simulation pipeline integrates biologically motivated flight dynamics, stochastic wind fields and species-specific behavioral parameters, enabling the generation of physically coherent and diverse trajectories without reliance on real-world data. The classification model, implemented as a bidirectional LSTM network, demonstrates strong learning behavior when trained on the generated dataset.

Although the study relies exclusively on synthetic data, the physics-based simulation pipeline allowed us to test the central hypothesis that motion trajectories alone can discriminate drones from birds in long-range scenarios. The results on synthetic test sequences indicate that trajectory-based classification is a promising complement to appearance-based methods for drone detection and avian behavior analysis. In future work, this hypothesis will be validated on real-world trajectories and operational surveillance data to quantify the domain gap between synthetic and real scenes and to refine the model using transfer-learning or hybrid real–synthetic training regimes. The modular design of the simulator will also facilitate extending the framework to additional species, environmental conditions and drone types.

Consequently, the proposed framework is compatible with real surveillance pipelines and is intended to be integrated with existing detection and tracking modules in future work.

## Figures and Tables

**Table 1:** Summary of parameters.

Symbol	Meaning	Unit	Typical Range
k_s_	speed adaptation rate	s^−1^	0.5–2
s*	preferred airspeed	m∙s^−1^	10–25
β_tw_	tailwind sensitivity	-	0.1–0.4
σ_s_	speed noise amplitude	m∙(s√s)^−1^	0.3–0.8
k_ϕ_	roll response rate	s^−1^	2–6
k_g_	goal-seeking strength	-	0.8–3.0
k_h_	altitude control strength	-	0.015–0.03
k_w_	wind compensation strength	-	0.3–2.0
k_a_	obstacle avoidance strength	-	2.0–4.0
ϕ_max_	maximum bank angle	rad	30–75°
σ_ϕ_	roll noise amplitude	rad∙(√s)^−1^	0.05–0.12
σ_a_	lateral acceleration noise amplitude	-	0.4–1.0
σ_u_	heading direction noise amplitude	-	0.06–0.1

**Table 2:** Model architecture.

Property	Value
input dimension	x ∈ R^3^ (it represents x, y and z)
hidden dimension	64
number of LSTM layers	2
bidirectional	True
dropout	0.2
output layer	fully connected (linear) layer mapping 128 hidden units (64 × 2 directions) to a single scalar
activation	sigmoid function for per-frame probability output between 0 and 1

**Table 3:** Probabilities per step.

Step	Probability
1	0.8733
2	0.9084
3	0.9230
4	0.9301
5	0.9340
6	0.9362
7	0.9375
8	0.9384
9	0.9390
10	0.9395
11	0.9398
12	0.9402
13	0.9405
14	0.9409
15	0.9412
16	0.9416
17	0.9419
18	0.9422

## Data Availability

The original contributions presented in this study are included in the article. Further inquiries can be directed to the corresponding author.
